# Breathing-phase selection for gated proton therapy in lung cancer towards temporally targeted ultra-high dose rate delivery

**DOI:** 10.1016/j.phro.2026.101065

**Published:** 2026-08-17

**Authors:** Simone N. Visser, Krista C.J. van Doorn-Wink, Koen F. Crama, Coen R.N. Rasch, Steven J.M. Habraken

**Affiliations:** a Department of Radiation Oncology, Leiden University Medical Center, Leiden, The Netherlands; b Holland Proton Therapy Center, Delft, The Netherlands

**Keywords:** Ultra-high dose rate, FLASH, Proton therapy, Lung cancer, Motion mitigation

## Abstract

**Background and purpose:**

Ultra-high dose rate (UHDR) irradiation has been demonstrated to reduce normal tissue damage compared to conventional dose rates, while maintaining tumor response (FLASH-effect). UHDR could potentially freeze intra-fraction breathing motion, enabling margin reduction for moving tumors when accurately timed. Targeting the optimal breathing-phase could reduce organ-at-risk (OAR) dose and side effects. In this treatment planning study, the optimal phase(s) for UHDR proton therapy were identified and potential benefits were evaluated.

**Materials and methods:**

Twenty lung cancer patients, previously treated with 66 GyE/24 or 60 GyE/30 fractions, were included. Four-dimensional computed tomography (4D-CT) scans with clinical target and OAR delineations were used to create new treatment plans for individual 4D-CT phases, one-phase plans (OPP) and multiple-phase plans (MPP). Clinically relevant dose-volume parameters and normal tissue complication probabilities (NTCP) were evaluated.

**Results:**

Phase-targeted proton therapy (PTPT) significantly reduced OAR dose. The largest reductions were achieved with OPP, while MPP showed smaller reductions. With OPP, mean lung dose (mean: −0.7 GyE, range: −1.7 to 0.3 GyE), mean heart dose (mean: −0.4 GyE, range: −1.4 to 0.5 GyE), and mean esophagus dose (mean: −0.9 GyE, range: −6.0 to 0 GyE) were reduced, with most reductions in the 0%, 40%, and 70% phases, respectively. NTCP values indicated reduced complication probabilities across all phases, with additional gains for optimal phases.

**Conclusions:**

With PTPT, OAR dose may be reduced with potential clinical benefit across all phases. The optimal phase depended on the endpoint, suggesting patient-specific phase targeting could further improve outcomes. Future research should address phase targetability, residual variation and required robustness.

## Highlights

• Phase-targeted proton therapy spared 16.8% (range 1.3–39.1%) of irradiated volume.
• Mean lung dose was reduced by 0.7 GyE (up to 1.7 GyE), most in full inspiration.
• Mean heart dose was reduced by 0.4 GyE (up to 1.4 GyE), most in expiration.
• Mean esophagus dose was reduced by 0.7 GyE (up to 6.0 GyE), optimal phase varied.
• Normal tissue complication probabilities were reduced for three side effects.

## Graphical abstract

**Figure:**
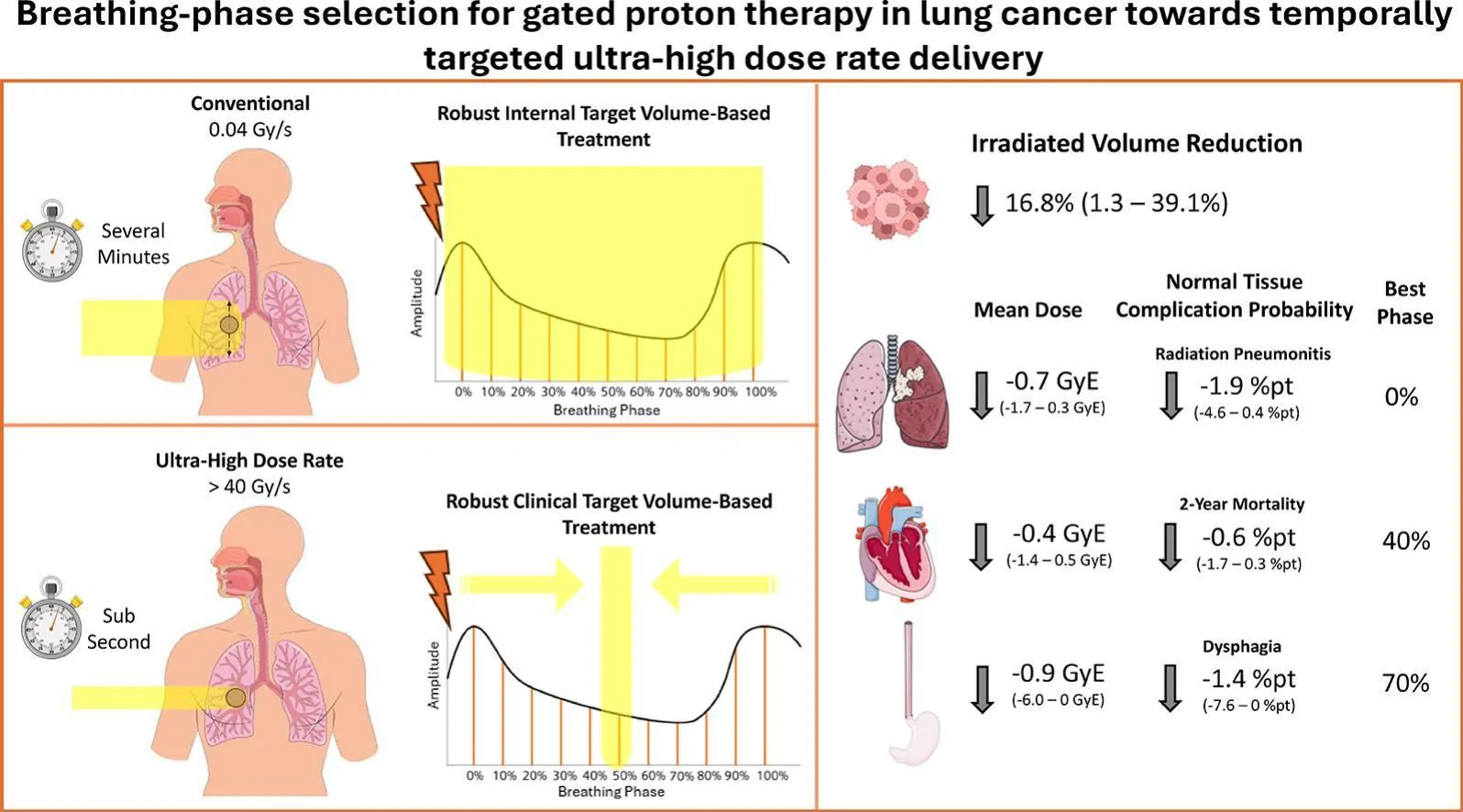

## 1 Introduction

Lung cancer is the leading cause of cancer-related deaths worldwide, with over 14,000 new cases diagnosed annually in the Netherlands and generally poor survival outcomes [1], [2]. Radiotherapy is commonly used in lung cancer treatment, either alone or combined with other treatments (e.g. chemotherapy), depending on disease stage, patient and tumor characteristics. In the Netherlands, radiotherapy is conventionally delivered with photons. Only patients who are expected to have significant benefit from proton therapy (PT), in terms of side effects, are referred to a proton center. With PT, radiation can be delivered more precisely to the tumor due to its characteristic dose–depth profile (the Bragg Peak), improving conformity and lowering integral dose [3], [4]. This may reduce side effects; however, not all patients experience a clinically significant benefit of PT compared to photons. Patient selection is based on a normal tissue complication probability (NTCP) model-based approach, comparing photon and proton plans in terms of the NTCP differences (ΔNTCP) [5].

Ultra-high dose-rate PT (UHDR-PT) has gained interest through the discovery of the FLASH-effect [6]. This effect, triggered by an UHDR of at least 40 Gy/s, reduced radiation damage to healthy tissue while preserving tumor response compared to conventional dose rates. It is demonstrated in several preclinical studies on various endpoints, including lung fibrosis [6], [7], [8], [9], [10]. UHDR-PT also enables sub-second dose delivery [11], offering opportunities for (time) gated treatment of moving targets, reducing the impact of respiration-induced target motion. While gating is well established with photons, UHDR-PT opens this possibility for protons, allowing a shift from planning over the full breathing cycle, where tumor motion is accounted for using an internal target volume (ITV) or mid-ventilation approaches [12], towards planning on a specific respiratory phase. This reduces the interplay effect [13] and therefore the necessity for repainting [14], which, combined with machine requirements on minimal pencil beam weight, may compromise plan quality in terms of dose [15]. With this approach, a reduction in irradiated volume - and thereby dose to healthy tissue - may be achievable, in addition to the proposed biological advantages of FLASH. However, the degree of organ-at-risk (OAR) sparing is expected to depend on the delivery phase, as target and OAR positions vary across phases [16].

The primary objective of this study was the identification of the optimal phase for gated PT, exploring potential improvements in treatment outcomes based on dose-volume parameters and NTCP-model predictions. Intensity-modulated proton therapy (IMPT) plans were generated on individual breathing-phases and analyzed to determine a phase, or range of consecutive phases, for which OAR dose was minimized while adequate tumor coverage was maintained, which may contribute to future UHDR-PT applications for lung cancer.

## 2 Materials and methods

### 2.1 Patient data

This retrospective study used four-dimensional computed tomography (4D-CT) scans from twenty consecutive lung cancer patients who underwent IMPT on a Varian ProBeam system at the Holland Proton Therapy Center (HPTC) between September 2022 and October 2023. The data was obtained from the HPTC research database and included only patients who had provided written informed consent for the use of their data for research. The database was reviewed by the Medical Research Ethics Committee Leiden–The Hague–Delft (reference number P18.053), which issued a statement of no objection. Patients were treated with a total prescribed dose of 60 or 66 GyE administered in 30 or 24 fractions. Patients received either radiotherapy alone or in combination with sequential or concurrent chemotherapy. Patients with a breathing amplitude <20 mm were included. Patient and tumor characteristics are shown in Table 1.

**Table 1:** Patient and tumor characteristics.

Characteristics	N
Total Patients	20
Males	9
Females	11

Mean age (in years)	65 (38–85)
Tumor position	
Middle or lower lobe	13
Upper lobe	7

Histology	
NSCLC	17
SCLC	3

Mean tumor motion amplitude (in mm)	
Left-right	1.8 (0.2–3.4)
Anterior-posterior	2.5 (0.3–7.4)
Inferior-superior	7.1 (0.3–15.8)
Mean (primary) GTV volume	116.2 cm^3^ (5.3–550 cm^3^)

Abbreviations: NSCLC, non-small cell lung cancer; SCLC, small cell lung cancer; GTV, gross target volume.

### 2.2 Delineations

Each 4D-CT scan was acquired using an Anzai belt and reconstructed into ten respiratory phases using time-based binning. The phases were labeled 0% to 90%, from full inhale (0%) to full exhale (50%) to near full inhale (90%). ITV-based clinical treatment planning was performed on the average intensity projections (AIP). Per clinical protocol, tumor and OARs were delineated on the AIP and on a limited number of individual phases. Remaining delineations were created using RayStation's (RaySearch Laboratories, Sweden) hybrid deformable image registration tool [17], by propagating contours from the reference AIP to remaining phases. All delineations were reviewed by an experienced lung radiation oncologist.

### 2.3 Treatment planning

Frozen intra-fraction breathing motion was simulated by performing treatment planning on individual 4D-CT phases, referred to as phase-targeted proton therapy (PTPT). No motion-encompassing margins were used, resulting in clinical target volume (CTV) based treatment plans. For each patient, ten one-phase plans (OPPs) were generated using an automated treatment planning protocol based on a class solution, mimicking clinical treatment planning while eliminating clinical variation. The planning constraints and objectives for dose guidance can be found in Table S1 (Supplementary Material A). Three consecutive optimization rounds were performed with the clinically used treatment planning system (RayStation 2023b), using a Monte Carlo dose calculation engine. Uncertainties in patient alignment and proton range were accounted for through robust optimization, ensuring adequate target coverage and OAR sparing [18]. Following the institutional clinical protocol, OPP were optimized for 7 mm setup and 3% range uncertainty, and plan robustness was assessed by the voxel-wise minimum (VWmin) and voxel-wise maximum (VWmax) across 28 scenarios [19]. VWmin evaluated target coverage, whereas VWmax evaluated potential hot spots in the OARs and target. Subsequently, ten multiple-phase plans (MPP) per patient were generated by creating new treatment plans on three consecutive phases. MPPs were created by robust optimization including three adjacent phases, thereby accounting for potential deviations in beam delivery timing and gating accuracy.

**Fig. 1:**
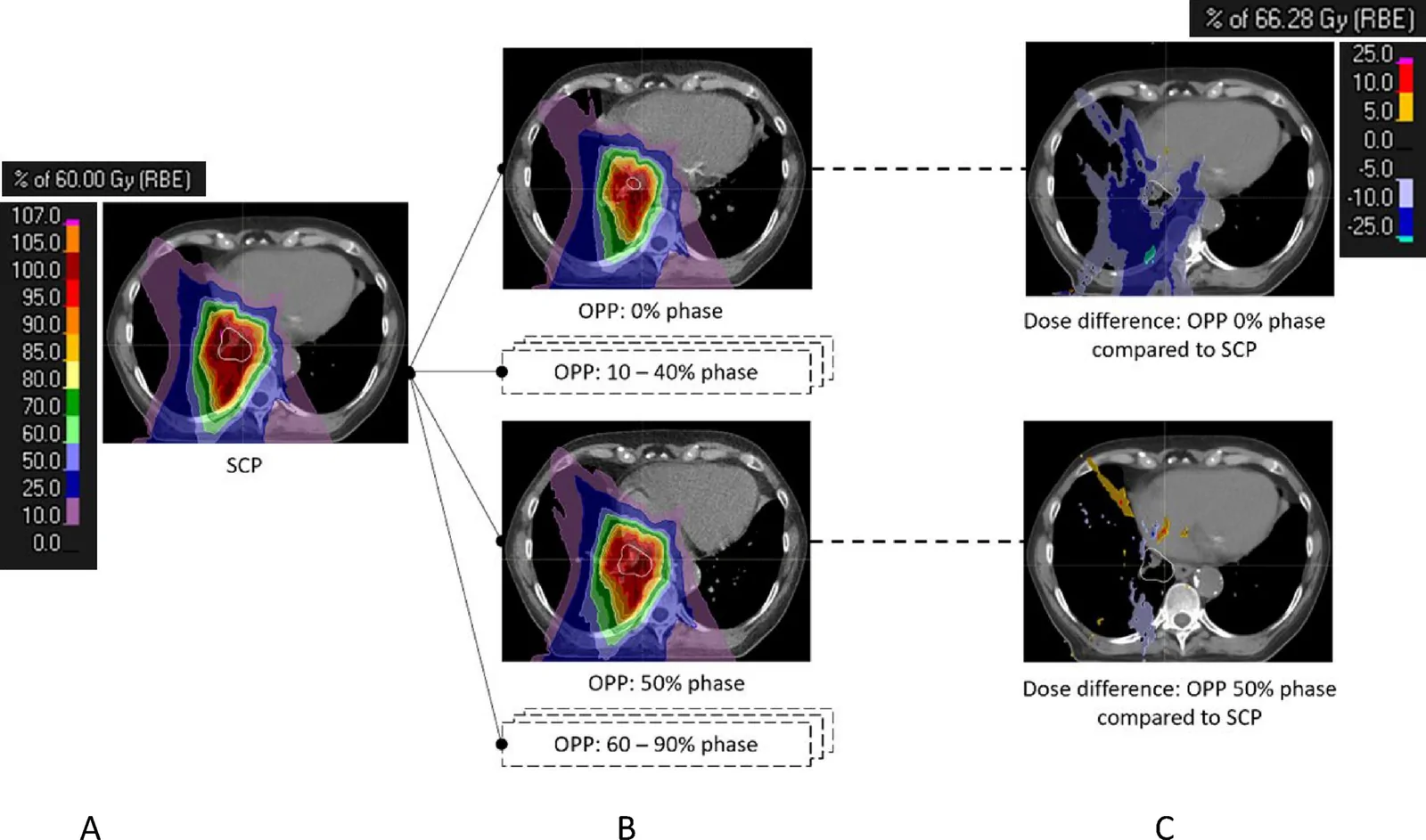
Example patient illustrating the planning methodology. The surrogate clinical plan (SCP) was created on the averaged intensity projection of the 4D-CT, one-phase plans (OPP) and multiple-phase plans (MPPs) were generated on the individual breathing-phases. Panel A shows dose distributions for the SCP (with the ITV as target). Panel B illustrates the OPPs on the individual phases with two examples at the 0% phase (full inspiration) and 50% phase (full expiration). Here, the target corresponds to the CTV. These examples demonstrate phase-specific differences in dose distribution compared to the SCP. Panel C depicts the dose differences between the SCP and the OPPs at 0% and 50%, expressed as percentage differences in maximum dose. Negative values (dark blue) indicate a 10–25% reduction in dose for the OPP compared to the SCP. (For interpretation of the references to color in this figure legend, the reader is referred to the web version of this article.)

To eliminate variability in clinical treatment planning, fully automated AIP-based reference plans, based on the same class solution as the OPP and MPP, were created as surrogate clinical plans (SCP) for comparison. However, the SCP followed the clinical approach for motion mitigation and were ITV-based. For three patients with a breathing amplitude >10 mm, layered repainting with five repaintings were used. In all plans, beam angle setup was identical to the clinical plans. Altogether, patient data comprised one SCP, ten OPPs, and ten MPPs per patient, yielding 420 treatment plans. The method is illustrated in Fig. 1.

### 2.4 Dose-volume analysis

Dose-volume histogram (DVH) parameters were analyzed. Tumor coverage was assessed by the percentage of the volume receiving ≥95% and ≥ 107% of the prescribed dose (V_95%_ and V_107%_). Lung metrics included mean lung dose (MLD) and the volume receiving ≥20 Gy and ≥ 5 Gy (V_20Gy_ and V_5Gy_). The MLD was expected to be lower in inspiration due to the larger lung volume, this volume-effect was additionally quantified by correcting the MLD for the phase-specific lung volume (Supplementary Material B). Heart metrics included mean heart dose (MHD), D_0.03cm_^3^ and V_30Gy_; esophageal metrics included mean esophagus dose (MED) and D_1.0cm_^3^. Differences in DVH parameters were assessed using paired *t*-tests (*p* < 0.05).

NTCP for radiation pneumonitis, dysphagia and two-year treatment-related mortality was calculated using models from the Dutch national PT indication protocol for lung cancer [20].

## 3 Results

CTV-based plans reduced target volume by an average of 25.1 cm^3^ (range: 2.5 to 80.3 cm^3^), which corresponded to a relative average reduction of 16.8% (range: 1.3 to 39.1%). Adequate target coverage was achieved in all plans, with CTV V_95%_ ≥ 98% and D_2%_ ≤ 107%. In the robustness evaluation, the plans adhered to clinical goals for CTV coverage in VWmin (V_95%_ ≥ 98%) and for serial OARs in VWmax.

**Fig. 2:**
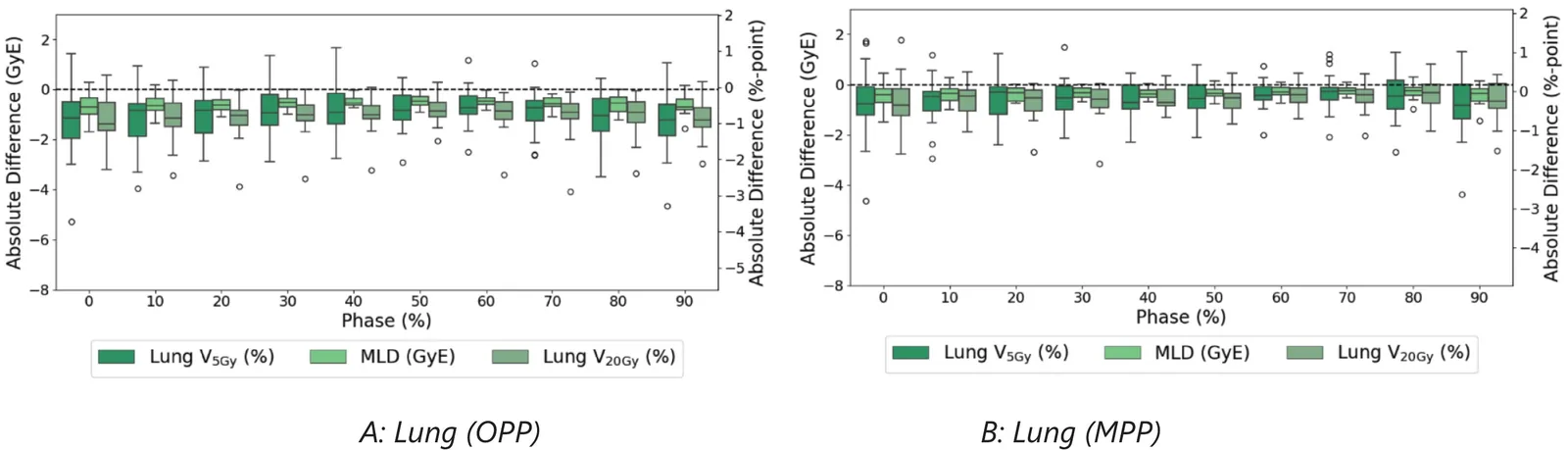
Absolute difference in dose (GyE) or volume (%-point) in dose volume histogram parameters for the lungs for the one-phase plans (OPP) compared to the surrogate clinical plan (SCP) (A) and for the multiple-phase plans (MPP) compared to the SCP (B). The dots indicate outliers. MLD, mean lung dose.

As shown in Fig. 2, the MLD decreased across all phases in OPP (all *p* < 0.001) and MPP (all p < 0.001). In OPP, the largest reduction in MLD was seen in the 0% phase (mean: −0.7 GyE, range: −1.7 to 0.3 GyE), compared to the population SCP median value of 11.7 GyE (range: 3.8 to 20.5 GyE). In MPP the optimal phase remained the 0% phase, (mean: −0.4 GyE, range: −1.5 to 0.5 GyE). The additional analysis on the effect of lung volume and breathing amplitude on the MLD is provided in Figs. S1–S4 (Supplementary Material B and C). Moreover, a significant reduction in lung V_20Gy_ in OPP was seen in all phases (all *p* < 0.001). The largest reduction was observed in the full inspiration 0% phase (mean: −1.2%-point, range: −3.2 to 0.6%-point), compared to the SCP median value of 22.4% (range: 7.3 to 41.3%). MPP also showed a significant reduction in lung V_20Gy_ (all *p* < 0.02) in all phases, with most reductions in the 30% phase (mean: −0.7%-point, range: −3.2 to 0%-point). A significant reduction in lung V_5Gy_ was observed in the OPP in all phases (*p* < 0.002, mean:-1.3%-point, range: −5.3 to 1.4%-point), compared to the SCP median value of 36.1% (range: 15.1 to 64.2%). In MPP, the reduction (mean: 0.7%-point, range: −4.6 to 1.7%-point) was only significant for phases 0–60% and 90% (*p* < 0.04).

**Fig. 3:**
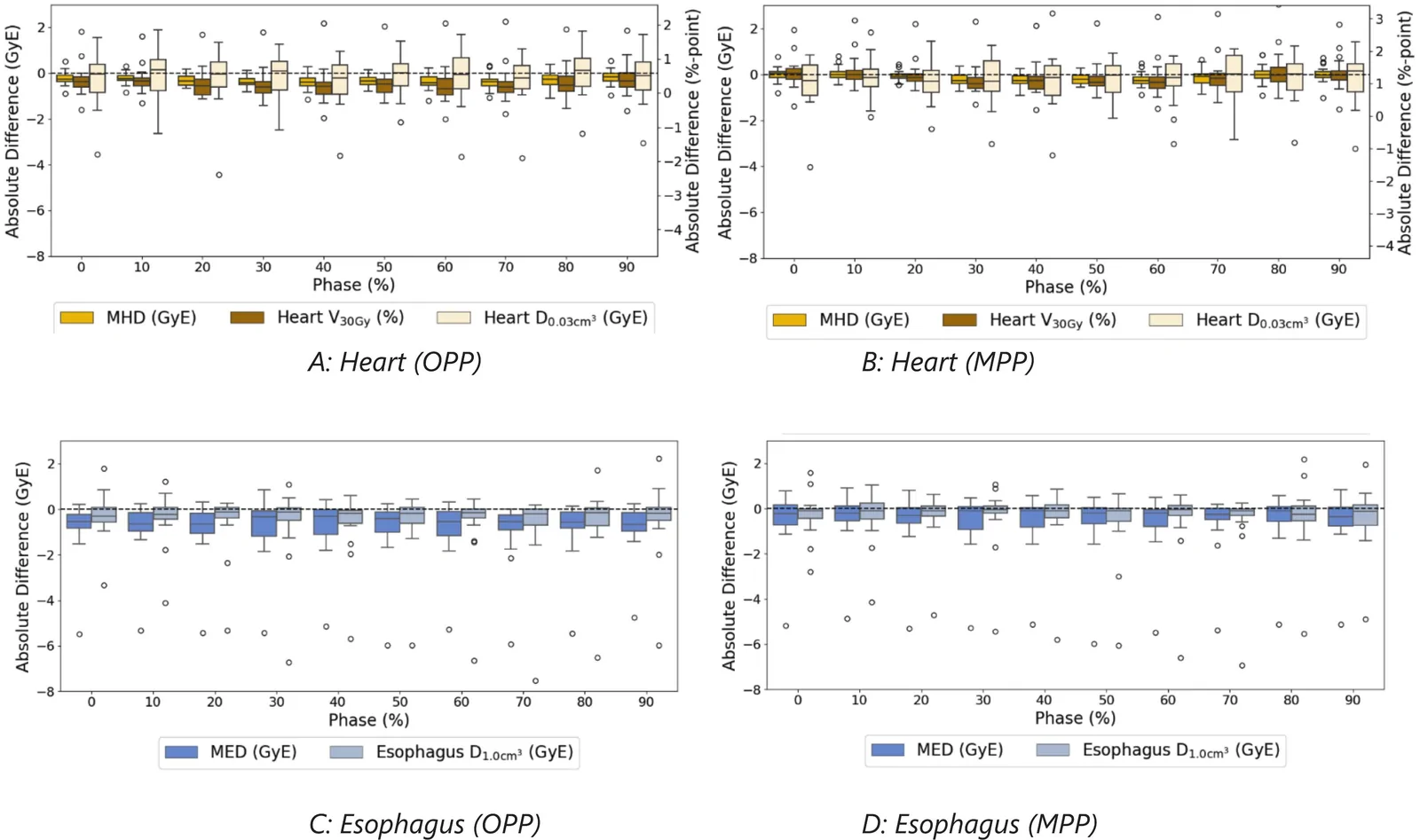
Absolute difference in dose (GyE) or in volume (%-point) in dose volume histogram parameters for the heart and esophagus for (A and C) the one-phase plans (OPP) compared to the surrogate clinical plan (SCP) and for (B and D) the multiple-phase plans (MPP) compared to the SCP. The dots indicate outliers. MHD, mean heart dose; MED, mean esophagus dose.

In Fig. 3, all phases showed significant reductions in MHD (all *p* < 0.02). The largest reduction was observed in the 40% phase (mean: −0.4 GyE, range: −1.4 to 0.5 GyE), compared to the SCP median value of 5.5 GyE (range: 0.0 to 12.3 GyE). With MPP, the MHD showed the largest reduction in the 40% phase again (mean: −0.2 GyE, range: −1.0 to 0.3 GyE). However, the reduction was only significant in the 40–60% phase (*p* < 0.05). A significant reduction in heart V_30Gy_ with OPP (mean: −0.5%-point, range: −1.9 to 2.2%-point) was observed in all phases (all *p* < 0.04), compared to the SCP median value of 7.2% (range: 0.0 to 16.5%). In MPP, the reduction (mean: −0.3%-point, range: −1.5 to 2.3%-point) was only significant for phases 30–60% (*p* < 0.03). A non-significant reduction in heart D_0.03cm_^3^ was observed in the OPP (mean: −0.4 GyE, range: −3.6 to 1.0 GyE), compared to the SCP median value of 64.5 GyE (range: 2.5 to 70.0 GyE). In MPP, heart D_0.03cm_^3^ was again non-significantly reduced (mean: −0.4 GyE, range: −4.0 to 0.8 GyE).

The MED in OPP showed a significant decrease in all phases (all *p* < 0.002), with the largest reduction in the 70% phase (mean: −0.9 GyE, range: −6.0 to 0 GyE), compared to the SCP median value of 20.0 GyE (range: 0.0 to 36.2 GyE). In the MPP, the MED decreased most in the 60% phase (mean: −0.6 GyE, range: −6.0 to 0.5 GyE), but was only significant for the 60%, 70% and 90% phases (*p* < 0.03). A significant reduction in esophagus D_1.0cm_^3^ with OPP (mean: −0.7 GyE, range: −7.5 to 0.2 GyE) was observed in the 20% and 40–70% phases (*p* < 0.05) compared to the SCP median value of 60.5 GyE (range: 0.1 to 65.9 GyE). In the MPP, the reduction (mean: −0.6 GyE, range: −6.9 to 5.9 GyE) was significant for phase 50% and 70% (*p* < 0.04).

**Fig. 4:**
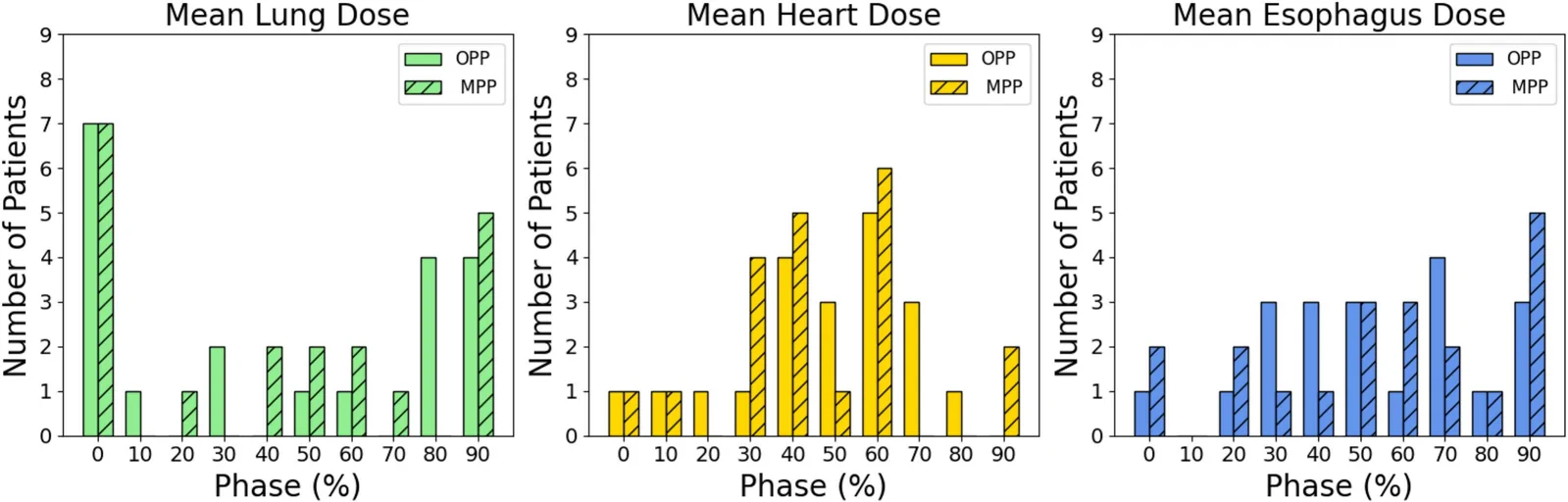
Optimal breathing-phases across 20 patients considering the mean lung dose, mean heart dose, and mean esophagus dose for one-phase plans (OPP) and multiple-phase plans (MPP). Bars represent the number of patients for whom a phase (0–90%) ranked the lowest mean organ-at-risk dose.

No significant differences were observed between the phases, indicating that no single optimal phase could be identified within this patient cohort. The optimal phase was therefore assessed per patient as shown in Fig. 4.

**Fig. 5:**
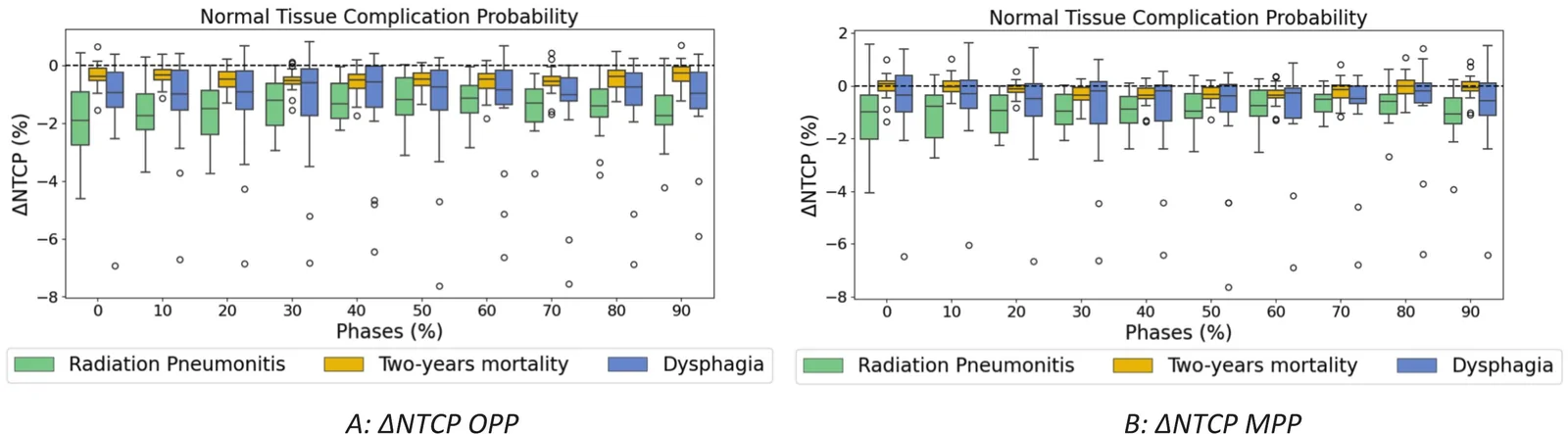
Difference in normal tissue complication probability (ΔNTCP) for three toxicities per phase for (A) one-phase plans (OPP) compared to the surrogate clinical plan (SCP) and (B) multiple-phase plans (MPP) compared to the SCP.

Fig. 5a shows that OPP led to decreased NTCP predictions, indicating a lower risk of complications. The risk of radiation pneumonitis was reduced most in the 0% phase (mean: −1.9%-point, range: −4.6 to 0.4%-point), compared to the SCP median value of 25.4% (range: 10.2 to 53.4%). For reducing two-year treatment-related mortality, the 40% phase was most beneficial (mean: −0.6%-point, range: −1.7 to 0.3%-point), compared to the SCP median value of 46.9% (range: 31.1 to 72.5%). The preferred phase for reducing dysphagia was the 70% phase (mean: −1.4%-point, range: −7.6 to 0%-point), compared to the SCP median value of 39.2% (range: 0.0 to 68.0%). The ΔNTCP differences for MPP are displayed in Fig. 5b. For radiation pneumonitis, the largest reduction was seen in the 0% phase (mean: −1.0%-point, range: −4.1 to 1.6%-point), while for two-year treatment-related mortality, the largest reduction was observed in the 60% phase (mean: −0.4%-point, range: −1.3 to 0.4%-point). For dysphagia, the 50% phase showed most reductions in NCTP prediction (mean: −1.1%-point, range: −7.6 to 0.5%-point).

## 4 Discussion

This study analyzed the dose-volume benefits of PTPT, which enabled statistically significant reductions in OAR dose, irradiated volume, and NTCP predictions. This was partly due to reductions of the target volume and partly to anatomical variation among different breathing-phases. No significant differences were observed between phases, so not one single optimal respiratory phase for PTPT could be identified. One contributing factor was that the phase associated with the largest dose reduction varied across endpoints. Inspiration yielded the lowest MLD, as expected given the larger lung volume. For MHD the largest reduction occurred in the expiration phases, whereas the preferred phase for MED was more variable.

The findings differ from previous studies applying phase-selection strategies, such as the mid-ventilation approach, designed to minimize dose distribution variations due to respiratory motion [12], [21]. Here, the aim was to identify the phase that, in the absence of breathing motion, yields the most favorable dose distribution.

Overall, tumor position, volume, and motion varied substantially between patients, contributing to interpatient differences in OAR dose. Lower- and middle-lobe tumors generally exhibit larger motion amplitudes than upper-lobe tumors [16], suggesting more benefit from PTPT in these patients. Tumors located far from the heart or esophagus resulted in minimal dose to these organs, whereas tumors in close proximity resulted in considerably higher and more variable OAR doses. Additionally, the heart is itself a moving organ with changes in volume occurring on a different timescale than respiratory motion, which is particularly relevant in UHDR treatment and should be considered when treating tumors near the heart, e.g., with combined respiratory and cardiac gating [22].

NTCP predictions emphasized the potential clinical gains through PTPT. The MLD reductions contributed to decreased NTCP for radiation pneumonitis and underscored the importance of reduced lung exposure [23]. Reductions in V_20Gy_ were likewise relevant, given its known association with radiation pneumonitis [24], [25]. Similarly, MHD reduction improves cardiac sparing, associated with lower heart toxicity and improved survival [26], [27]. Reduced MED suggested a lower risk of dysphagia, an important determinant of post-radiotherapy quality of life [28], [29]. Moreover, larger ∆NTCP differences may increase patient eligibility for PT referral.

Some limitations should be considered. All plans used a standardized class solution with clinical robustness margins, to generate reproducible and comparable plans without introducing variability from individualized planning choices. Yet, it remains uncertain whether these robustness margins are compatible with UHDR-PT. Breathing motion was assumed to be completely mitigated, whereas in a clinical setting, the residual respiratory motion should be identified and accounted for. Another limiting factor is the assumption of similar respiratory motion between a time-gated planning 4D-CT and the actual treatment, since substantial breathing variability is known to occur [30]. The MPP is intended to partially account for residual motion and uncertainties, although the magnitude of these uncertainties was not quantified here and needs further evaluation. The reductions seen with MPP were smaller than with OPP but remained substantial, with clear improvements compared with SCP.

Although the PTPT plans were not inherently UHDR-compatible, UHDR delivery conditions could be closely approximated, for example, through gating, breath-hold (BH) or assisted breathing techniques. The comparison study of Wei et al. showed little difference in dose to OARs between IMPT and FLASH plans in 10 lung cancer patients [31]. Furthermore, studies describe the use of range shifters and range compensators to adapt IMPT plans for FLASH treatment planning [32], [33]. The precise planning and treatment protocols for FLASH are actively researched, including fractionation, beam angle configuration and the use of transmission versus Bragg peak beams [34], [35], [36]. Machine requirements concerning dose and dose rate thresholds, beam-on time per field, target volume range, scanning/delivery speed, minimum MU per spot, and typical delivery time are also under investigation. The current study intentionally followed clinical practice as closely as possible since the treatment requirements for FLASH are still under development. This study demonstrated that phase selection may improve dose distributions and NTCP, future gated UHDR-PT strategies and potentially enabling PT for patients with breathing amplitudes >20 mm.

These findings are also relevant beyond FLASH and may contribute to the development of gated PT for lung cancer [37] since motion management remains a major challenge in radiotherapy [38], [39]. In photon therapy, motion mitigation techniques including BH, respiratory gating, assisted-breathing and tracking [3], [40], [41], effectively reduced treated volumes [42], [43], [44], [45], [46]. However, their direct translation to PT is limited due to fundamental differences in dose deposition [3]. Individualized margins are commonly used in PT, but opportunities exist to expand towards other motion mitigation strategies. Deep inspiration BH reduces radiation-induced side effects in the heart, lung and esophagus [47]. However, its applicability is limited, particularly for lung cancer patients who may struggle to maintain breath-holds. This may not pose a challenge with sub-second dose delivery, but it is challenging during longer procedures such as CT acquisition [48]. Gating, on the other hand, reduces the target volume requiring irradiation and minimizes dose degradation from tumor movement [49]. The drawback of gating is prolonged treatment time due to beam interruptions, but UHDR-PT has the potential to overcome this limitation. Combining gating with assistant breathing or having a sufficient surrogate could account for breathing irregularities. Overall, UHDR-PT could help the development of novel and more efficient motion mitigation approaches in PT.

UHDR-PT offers further benefits for lung cancer. Given current understanding of UHDR and radiation-induced lymphopenia (RIL), sub-second dose delivery could plausibly reduce the number of irradiated circulating lymphocytes [50], [51]. This would lower the likelihood of patients developing RIL. Supporting this, a simulation study on brain cancer by Galts et al. demonstrated that with FLASH the irradiated blood volume decreased with 35.1% to 40.9% and the depletion rate of lymphocytes was reduced by 69.2% compared to conventional IMPT [52].

As phase choice did not affect PTPT dose-volume outcomes, phase stability may be a relevant endpoint for future studies. Literature indicates that the expiration phase is generally the most stable part of the respiratory cycle [49], [53]. Yet, further research is needed to investigate phase stability and targetability in lung cancer patients undergoing PT.

Compared with conventional planning, PTPT showed statistically and potentially clinically significant differences across all phases, highlighting its potential to reduce OAR dose and NTCP in practice. The optimal phase depends on the endpoint, so patient-specific weighting could be used to select the optimal phase for a patient group or individual patient. These findings suggest a potential benefit of UHDR-PT for lung cancer and as a motion mitigation strategy. Future research should address possible variations in stability and targetability, as well as explore alternative approaches beyond or combined with UHDR for eliminating respiratory motion.

## CRediT authorship contribution statement

**Simone N. Visser:** Writing – original draft, Visualization, Validation, Methodology, Investigation, Formal analysis, Data curation, Conceptualization. **Krista C.J. van Doorn-Wink:** Writing – review & editing, Validation, Supervision, Resources, Methodology, Investigation, Funding acquisition, Data curation, Conceptualization. **Koen F. Crama:** Writing – review & editing, Resources, Methodology. **Coen R.N. Rasch:** Writing – review & editing, Supervision, Project administration, Methodology, Investigation, Funding acquisition, Conceptualization. **Steven J.M. Habraken:** Writing – review & editing, Visualization, Supervision, Project administration, Methodology, Investigation, Funding acquisition, Formal analysis, Conceptualization.

## Declaration of generative AI and AI-assisted technologies in the writing process

During the preparation of this work the author(s) used ChatGPT/Claude to assist with text refinement. After using this tool, the authors reviewed and edited the content as needed and take full responsibility for the content of the publication.

## Declaration of competing interest

The authors declare the following financial interests/personal relationships which may be considered as potential competing interests: This work is part of the “FLASH beyond doubt, and beyond” project, funded by Varian, a Siemens Healthineers Company, within the framework of the HollandPTC consortium R&D program - Varian research collaboration (HollandPTC-Varian 2020, grant number 2020016).
