# Supplementary material for: Breathing-phase selection for gated proton therapy in lung cancer towards temporally targeted ultra-high dose rate delivery
Source: Phys Imaging Radiat Oncol. 2026 Aug 17;40:101065. doi: 10.1016/j.phro.2026.101065 (PMC13544324; doi:10.1016/j.phro.2026.101065)
Supplement: Supplementary file 1 [file mmc1.pdf]

Breathing-phase selection for gated proton therapy in lung cancer towards temporally targeted ultra-high dose rate delivery

Supplementary Material A

Table S1: Constraints and planning objectives used for dose guidance.

ROI	Constraint / objective
ITV	$D_{98\%} \geq 95\%$<br>$D_{2\%} \leq 107\%$
CTV	$D_{98\%} \geq 95\%$<br>$D_{2\%} \leq 107\%$
Lungs-GTV	$D_{mean} \leq 20$ GyE<br>$V_{20Gy} \leq 35\%$
Esophagus	$D_{mean} \leq 34$ GyE<br>$D_{1.0cm^3} \leq 60$ GyE
Heart	$V_{30Gy} \leq 30\%$
Spinal cord core	$D_{0.03cm^3} \leq 50$ GyE
Spinal cord surf	$D_{0.03cm^3} \leq 60$ GyE
Skin	$D_{0.03cm^3} \leq 50$ GyE

***Abbreviations**: ITV, internal target volume; CTV, clinical target volume; GTV, gross tumor volume; Dmean, mean dose; Dx%, dose received by x% of the volume;* $D_{0.03cm^3}$ / $D_{1.0cm^3}$, *minimum dose to the most exposed 0.03/1.0cm*^{3}*; VxGyE, percentage of volume receiving ≥x GyE.*

Supplementary Material B

The mean lung dose (MLD) is the total lung dose divided by the lung volume. When targeting a single phase, as in the one-phase plans (OPP), the lung volume of that phase influences the MLD. Therefore, an additional analysis was performed to quantify this volume effect. Figure S1A shows the MLD for 20 patients across all phases; the inspiration phases yielded the lowest MLD.

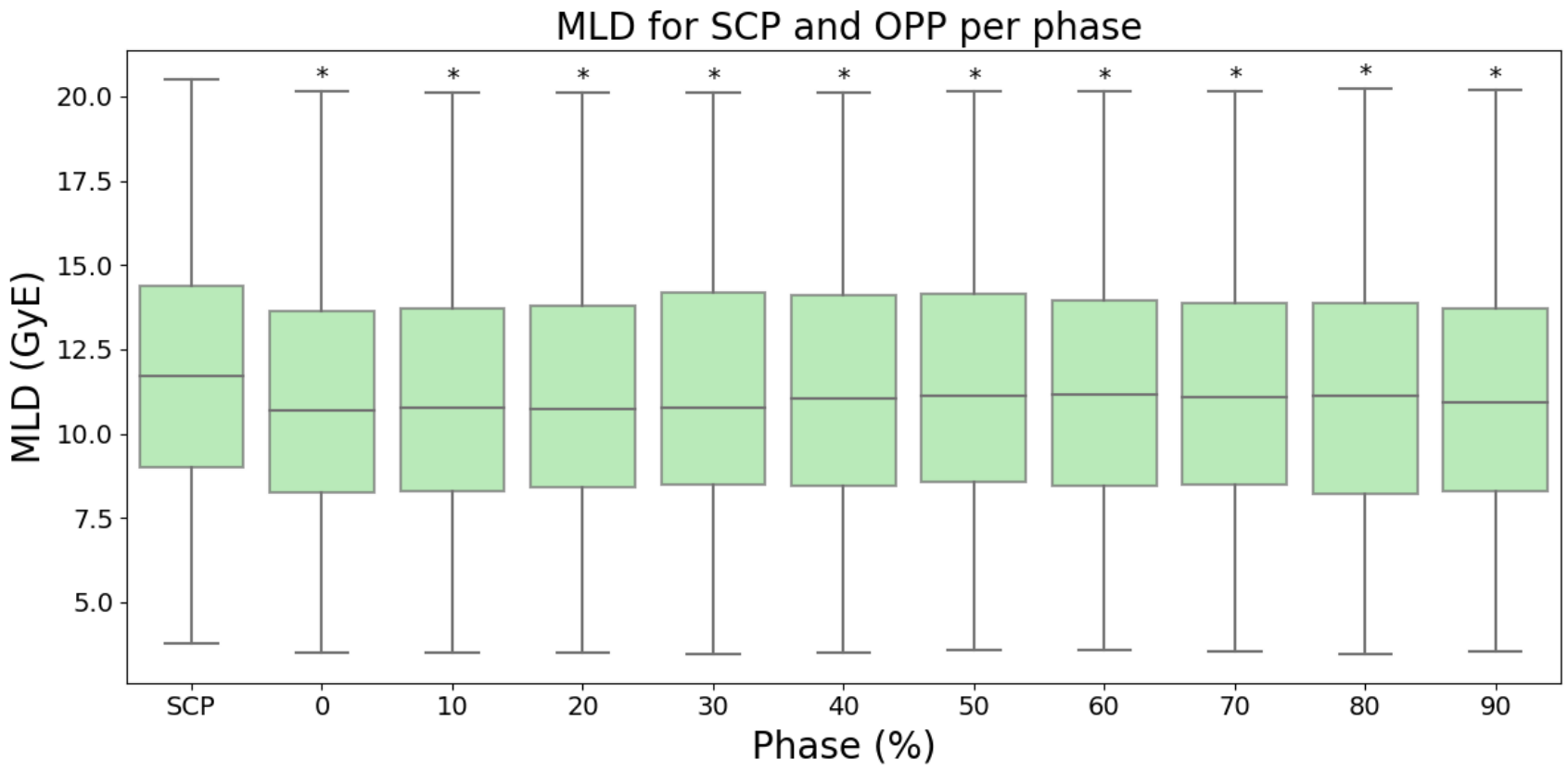

A: MLD for SCP and all phases

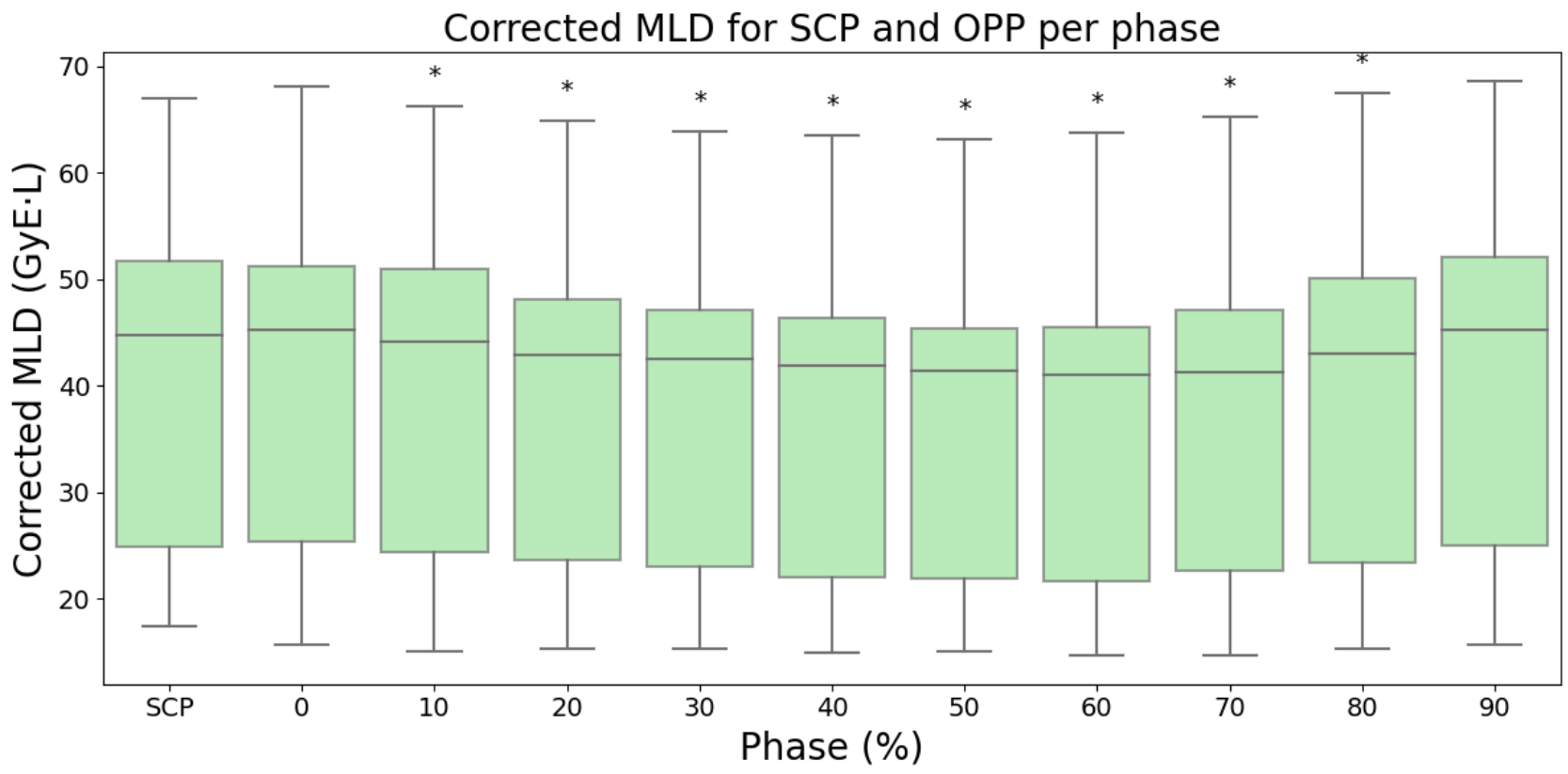

B: Volume-corrected MLD for SCP and all phases

Figure S1: Mean lung dose (MLD) and corrected MLD in the surrogate clinical plan (SCP) and all phases for one-phase plans (OPP). In both figures the single asterisk (*) indicates that the MLD or corrected MLD is significantly lower (all $p < 0.05$) in the OPP.

In Figure S1B, the MLD is corrected for the volume-effect. Because the MLD is a volume-normalized quantity, a larger lung volume in a given phase lowers the MLD even when the absolute deposited dose is comparable. To remove this dependency, each patient's phase specific MLD was multiplied by the corresponding lung volume of that phase. For example, the MLD in phase 0% was multiplied by the lung volume in phase 0%, the MLD in phase 10% by the lung volume in phase 10%, and so on for every phase. This correction was applied to each phase and each patient individually. The volume-corrected MLD shows a reverse effect: the expiration phases now yield the lowest MLD, while the inspiration phases yield even a higher MLD than the surrogate clinical plans (SCP). This confirms that the larger lung volume in the inspiration phases strongly affects the MLD, which should be considered when selecting an optimal phase if the mean dose to the lungs is considered as an endpoint.

Supplementary Material C

Phase targeted proton therapy (PTPT) leads to greater volume reduction in patients with larger breathing amplitudes, when switching from internal target volume (ITV) based to clinical target volume (CTV) based plans because breathing motion is incorporated in the ITV. This significant correlation is shown in Figure S2 (r = -0.62, p = 0.004).

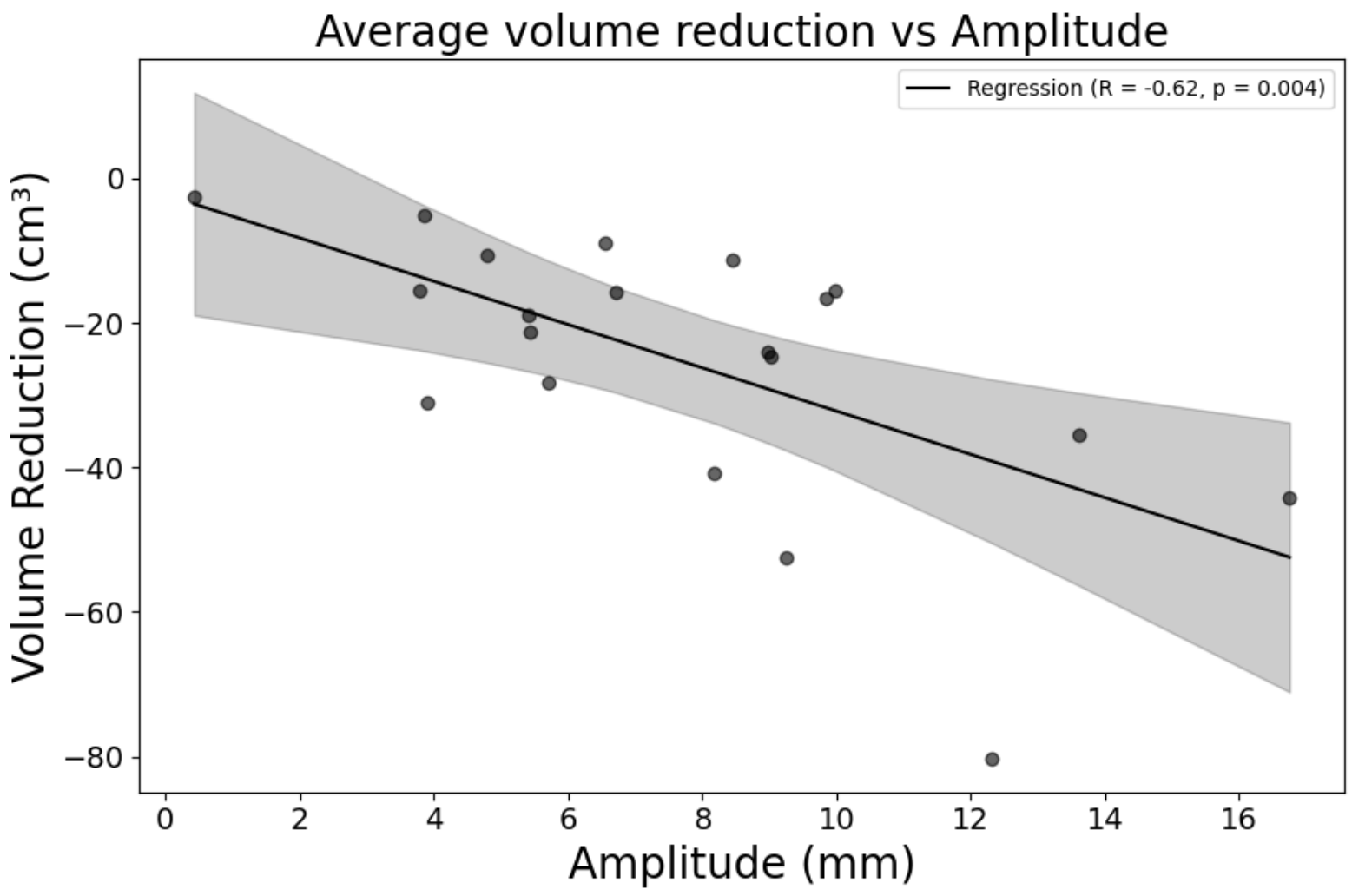

Figure S2: Correlation between breathing amplitude and reduced absolute volume for all patients, including regression line and Pearson' r value.

Figure S3 shows a significant correlation between larger tumor motion (amplitude) and reductions in mean lung dose (MLD) with one-phase plans (OPP) (r = -0.55, p = 0.012). On average, patients with more tumor motion received less dose (in GyE). Figure S4 shows that there is no significant correlation between larger amplitudes and reduction in MLD with multiple-phase plans (MPP) (r = -0.23, p = 0.330).

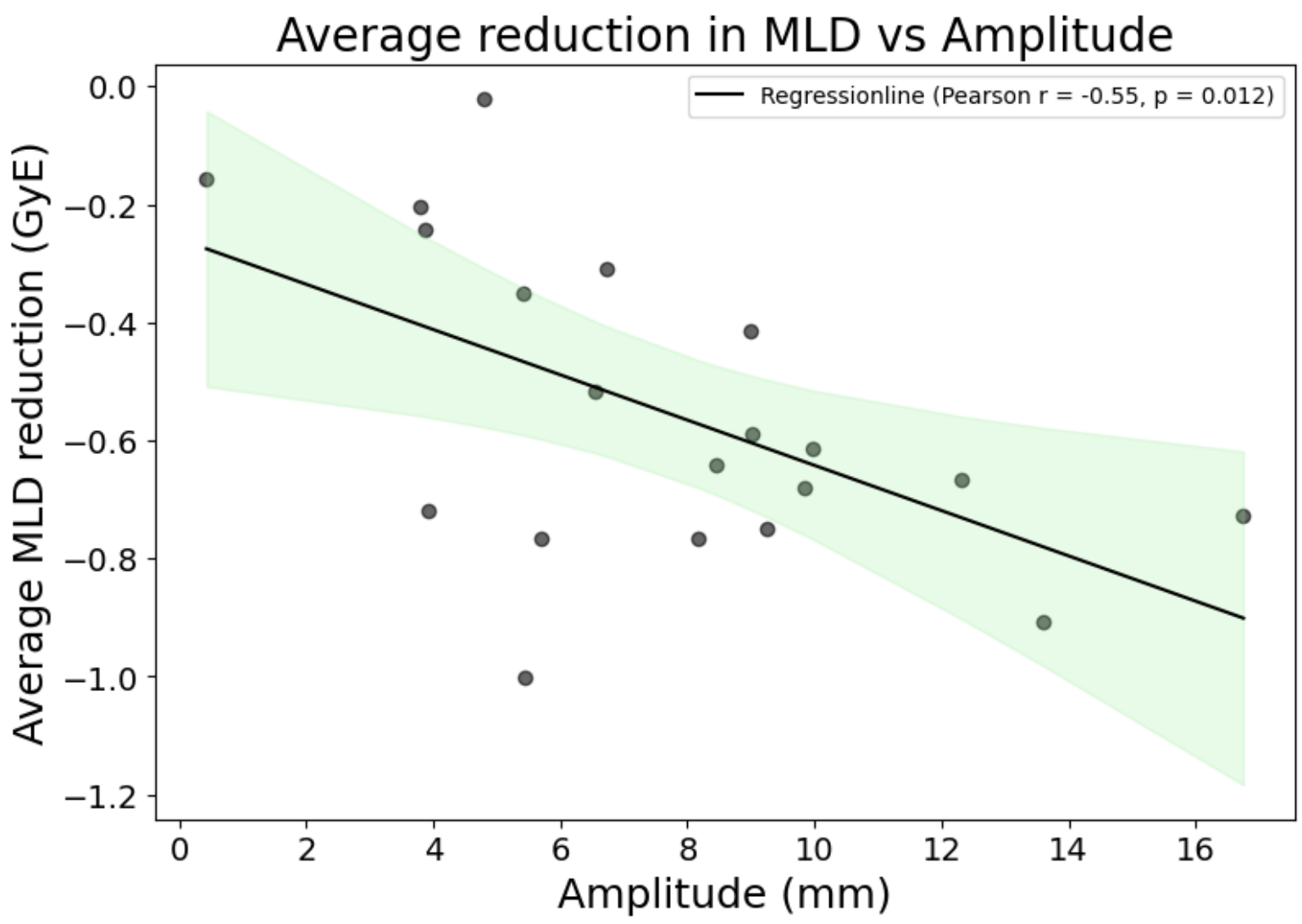

Figure S3: Correlation between amplitude and average gain in mean lung dose with one-phase plans, including regression, 95% confidence interval and Pearson' r value.

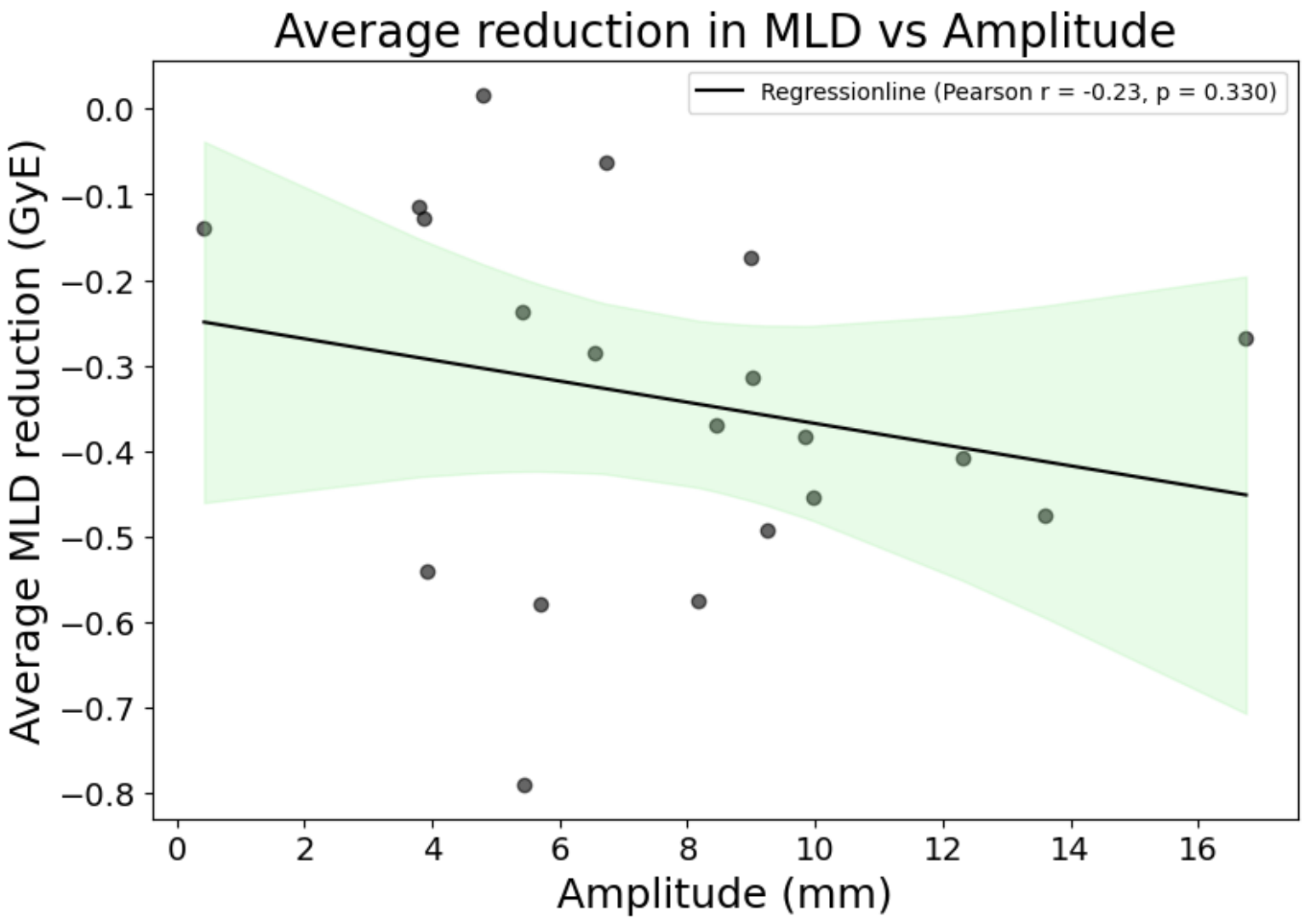

Figure S4: Correlation between amplitude and average gain in mean lung dose with multiple-phase plans, including regression, 95% confidence interval and Pearson' r value.
